# Distribution and habitat use of red panda in the Chitwan-Annapurna Landscape of Nepal

**DOI:** 10.1371/journal.pone.0178797

**Published:** 2017-10-11

**Authors:** Damber Bista, Saroj Shrestha, Peema Sherpa, Gokarna Jung Thapa, Manish Kokh, Sonam Tashi Lama, Kapil Khanal, Arjun Thapa, Shant Raj Jnawali

**Affiliations:** 1 Red Panda Network, Kathmandu, Nepal; 2 WWF Nepal, Kathmandu, Nepal; 3 Freelance Researcher, Kathmandu, Nepal; 4 Small Mammals Conservation and Research Foundation, Kathmandu, Nepal; Sichuan University, CHINA

## Abstract

In Nepal, the red panda (*Ailurus fulgens)* has been sparsely studied, although its range covers a wide area. The present study was carried out in the previously untapped Chitwan-Annapurna Landscape (CHAL) situated in central Nepal with an aim to explore current distributional status and identify key habitat use. Extensive field surveys conducted in 10 red panda range districts were used to estimate species distribution by presence-absence occupancy modeling and to predict distribution by presence-only modeling. The presence of red pandas was recorded in five districts: Rasuwa, Nuwakot, Myagdi, Baglung and Dhading. The predictive distribution model indicated that 1,904.44 km^2^ of potential red panda habitat is available in CHAL with the protected area covering nearly 41% of the total habitat. The habitat suitability analysis based on the probability of occurrence showed only 16.58% (A = 315.81 km^2^) of the total potential habitat is highly suitable. Red Panda occupancy was estimated to be around 0.0667, indicating nearly 7% (218 km^2^) of the total habitat is occupied with an average detection probability of 0.4482±0.377. Based on the habitat use analysis, altogether eight variables including elevation, slope, aspect, proximity to water sources, bamboo abundance, height, cover, and seasonal precipitation were observed to have significant roles in the distribution of red pandas. In addition, 25 tree species were documented from red panda sign plots out of 165 species recorded in the survey area. Most common was *Betula utilis* followed by *Rhododendron spp*. and *Abies spectabilis*. The extirpation of red pandas in previously reported areas indicates a need for immediate action for the long-term conservation of this species in CHAL.

## Introduction

The red panda (*Ailurus fulgens*) is the sole representative of the monotypic family *Ailuridae* [1,2] and a globally endangered species [3]. Red pandas inhabit eastern Himalayan temperate broadleaf forests with a bamboo understory and subalpine areas within a preferred altitudinal range of 2400–3900 m [4, 5]. This animal occupies middle elevations of 2800–3000 m in eastern Nepal and middle to high elevations of 2800–3900 m in central Nepal [5,6]. Sightings from lower elevations have also been recorded, e.g. at 2210 m in Ilam, eastern Nepal (first author, pers. obs., 2013). Red panda prefers the habitat with proximity to water sources (within 100–200 m); tree canopy cover (>30%); bamboo cover (>37%) and bamboo height (2.9 m) [4,6,7,8]. Similarly, the habitat with gentle to steep slopes with fallen logs, tree stumps, and snags, and the north, north-west and south-west aspects have been found to be highly preferred by the red panda [4,5,8,9]. Nepal holds the westernmost record from Kalikot district, western Nepal [10].

Red panda’s major diet is comprised of bamboo leaves and shoots, which account for more than 83% of the total food types [7]. These animals use elevated objects, such as shrub branches, fallen logs, or tree stumps to access bamboo leaves [11]. Since bamboo has very low caloric value, the red pandas spend nearly 56% of their overall time budget on eating [7]. Normally, they become more active in the early morning and evening hours while they spend rest of time for aging and sleeping on the tree branches or in tree hollows during the day [7]. Red panda is solitary species during non-breeding season and found in small groups during breeding season [12]. Red pandas are able to reproduce at around 18 months of age, and are fully mature at two to three years. Both sexes may mate with more than one partner during the mating season from mid-January to early March [13]. After a gestation period of 112 to 158 days [1] red panda gives birth in the early summer months from June to August to small litters of one to two young, occasionally three to four in a litter [14]. The young leave their mother to become independent at about 8 months of age, when the mother begins new breeding season [14].

A very limited number of studies on the red panda have been carried out so far, most of them focused on Langtang National Park (LNP) in the Chitwan-Annapurna Landscape (CHAL) in Nepal. Four ecological studies using radio-collars were conducted from 1984 to 2011, one of which was carried out in LNP by radio collaring six individuals from 1985 to 1987 [15]. This study revealed that red pandas are habitat specialists and prefer fir-jhapra (*Himalayacalamus falconeri*) forests between 2800 m and 3900 m in elevation. Another study carried out by Yonzon & Hunter [16] estimated around 68 km^2^ of habitat populated by 24 individuals probably isolated into four different populations.

A recent Population and Health Viability Assessment workshop produced the most robust information on red panda status in Nepal including CHAL [17]. This study estimated the total potential red panda habitat available in Nepal is 2652 km^2^, much lower than previous estimations of 20,400 km^2^ [18] and 17,400 km^2^ [19]. Likewise, estimates of the total red panda population in Nepal have ranged from 237 to 1061 individuals [17]. This population has been further divided into 11 sub-populations with two sub-populations (Annapurna-Manaslu and Langtang) falling within the present study area of CHAL. The 2010 Population and Health Viability Assessment workshop estimated a red panda population of around 21 individuals with a potential population of 84 individuals in six districts and four Protected Areas (PAs) distributed over 141.06 km^2^ of confirmed and an additional 644.85 km^2^ of potential habitat in CHAL. Finally, more specific studies in CHAL have focused on the relationship between livestock herding, tourists and red pandas [20] habitat and feeding behavior [21,22] and distribution and conservation threats [23]. Although these studies produced important information on distribution, habitats, feeding behavior, home range and habitat preferences of red pandas, they were very much localized and even few other large scale studies carried out in the past were based on very limited field work. All those studies barely provide accurate information of red panda from this landscape which is critical for devising long term conservation plan for this species. Thus, based on extensive field work, the present study aims to document and describe the distribution, abundance and habitat use of red pandas in the CHAL to create the first-ever broad-scale scientific basis for the promotion of red panda conservation efforts in this region.

## Materials and methods

This study also comprised consultation with local people which was approved by the Scientific Advisory Committee of Red Panda Network before the field survey began. The respondents were directly met during the survey and briefly informed about the nature of study to get their consent before interviewing them, and only those who were ready to answer were interviewed (n = 242). Their pseudonyms were recorded on the datasheet and data was tabulated in spreadsheet by a volunteer to maintain anonymity of the respondents.

**Fig 1:**
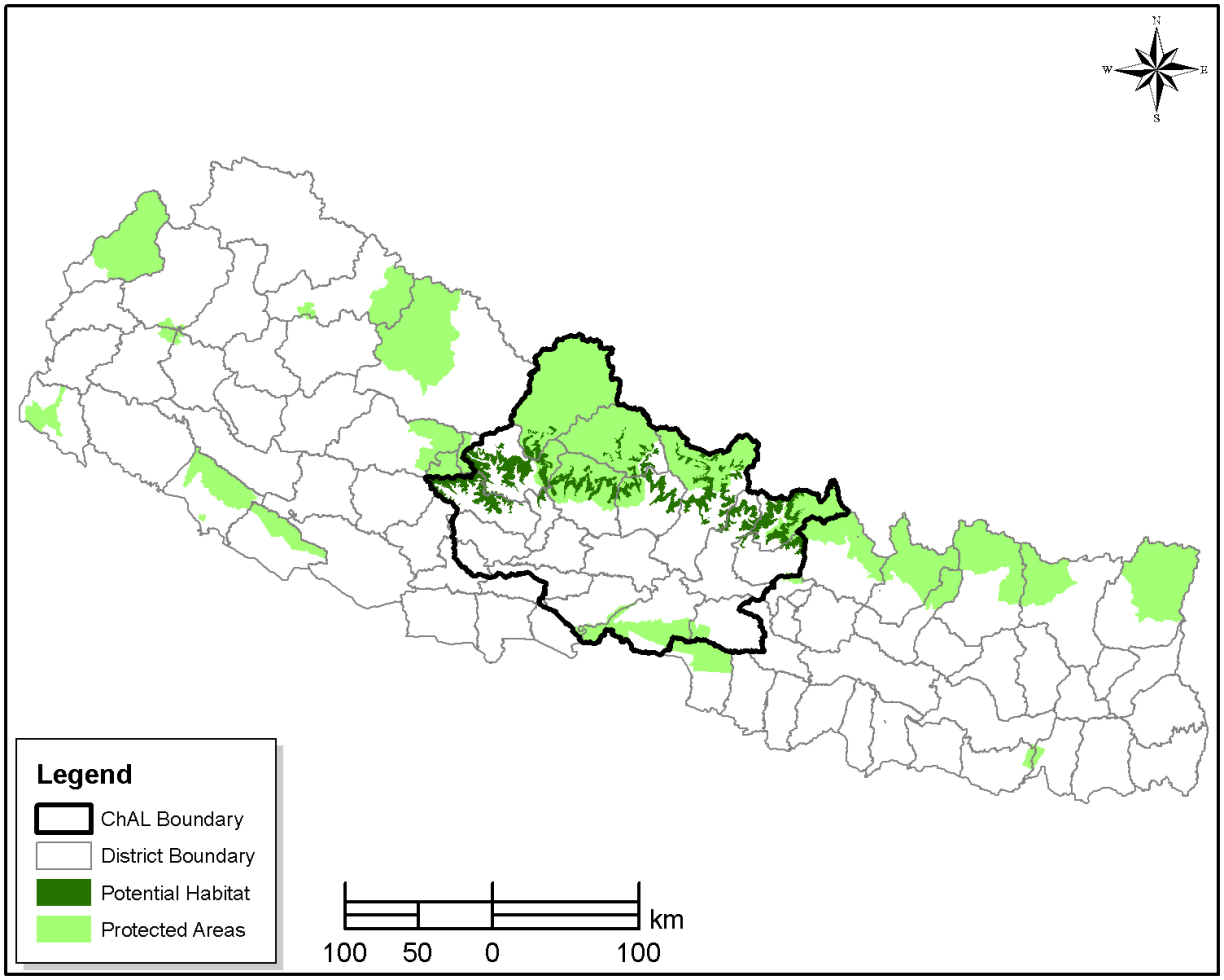
Chitwan Annapurna Landscape (CHAL).

The CHAL is located in central Nepal, covering an area of 32,057 km^2^, with elevation ranging from 200 m to 8091 m. This landscape covers all or part of 19 districts and seven PAs. Red pandas have been documented only in Langtang National Park (LNP), Manaslu Conservation Area, Annapurna Conservation Area (ACA), and Dhorpatan Hunting Reserve [17,21,23]. These studies have also reported sightings in Nuwakot, Rasuwa, Gorkha, Manang, Myagdi, and Baglung districts. The study area covers 11 districts in CHAL: Nuwakot, Rasuwa, Baglung, Gorkha, Myagdi, Manang, Mustang, Kaski, Lamjung, Dhading and Parbat (Fig 1).

The CHAL also offers links between Gaurishankar Conservation Area [24] in the east and Dhorpatan Hunting Reserve [21,23] in the west with the confirmed presence of red pandas. Based on anecdotal records and previous studies, red pandas have been confirmed in the Nuwakot, Rasuwa, Baglung, Gorkha, Myagdi and Manang districts, while the remaining 5 districts contain only potential habitats without confirmed records [17].

### Data collection and analysis

#### Distribution and abundance

ICIMOD land use map (http://geoportal.icimod.org/) was used to identify potential red panda habitat on the basis of an elevation range between 2200 m [25] and 4000 m, [5] and forest cover including fir, rhododendron, birch, alpine scrub [5], oak, broad-leaf deciduous, broad-leaf conifer, and coniferous tress [5,6].

**Fig 2:**
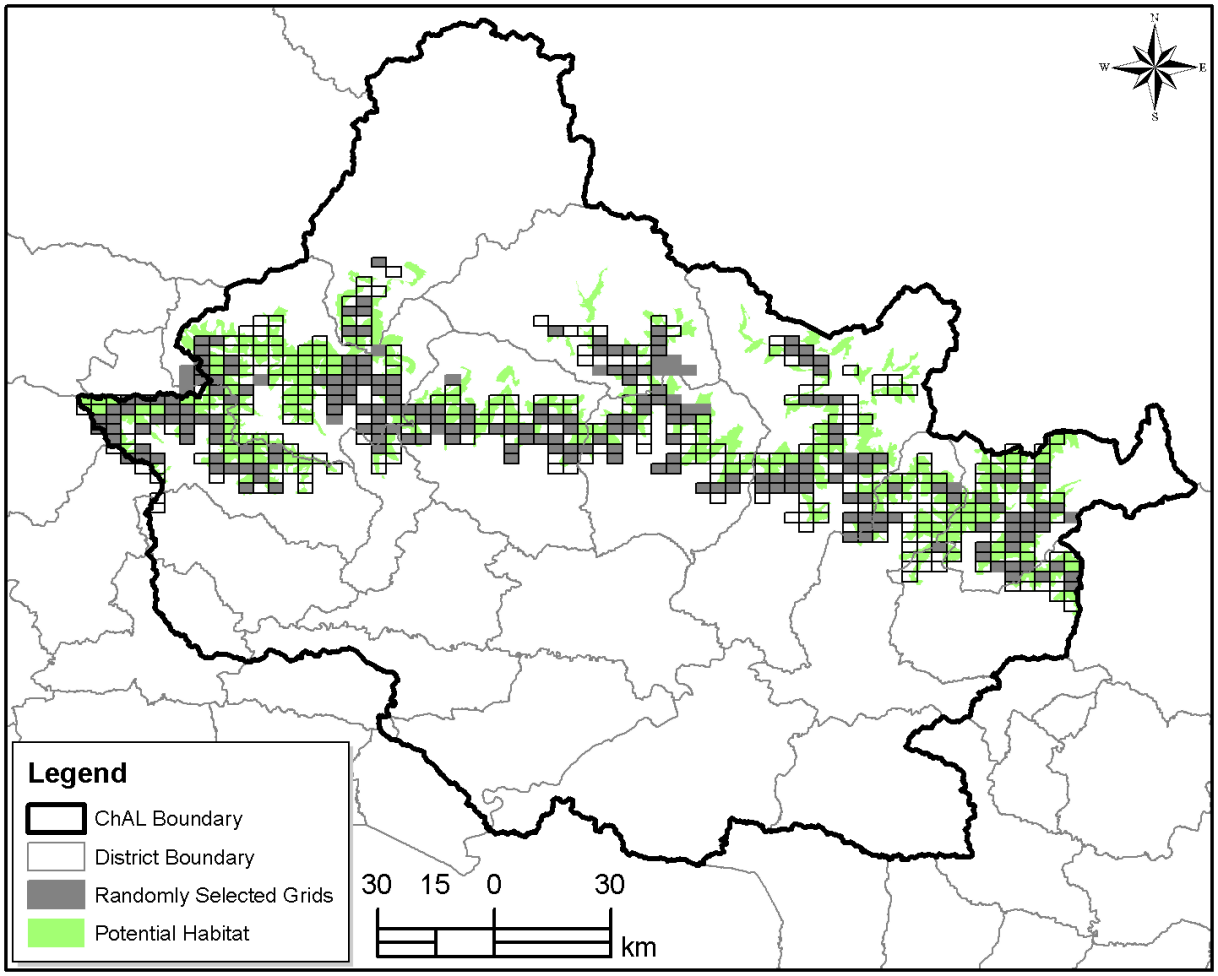
Grid distribution for field survey in CHAL.

This helped identify the potential red panda habitat in the CHAL. The identified habitat was overlaid with grids of 9.6 km^2^ (red panda’s maximum home range recorded in LNP) [26]. After this, 50% of these grids were selected (Fig 2) by using Geospatial Modeling Environment built in ArcGIS 10.2 version. Each selected grid was further sub-divided into 6 sub-grids each with an area of 1.6 km^2^ to ease the data collection. Finally, 50% of sub-grids i.e. 3 sub-grids in each grid were randomly selected. In this way, altogether 240 grids were covered [27]. All the available transects with an average length of 1 km at an interval of 100 m contour available within each sub-grid were sorted and their start and end points were recorded and loaded in GPS to ease in tracking those transects in field. However, the number of transect surveyed in a grid ranged from 1 to 4 based on the elevation gradient and accessibility of terrain. We traversed 332.68 km transect length during 2832 working hours in search of red panda sightings or presence signs (droppings, feeding signs, scratch marks and foot prints) and fixed each record by GPS (Garmin eTrex 10). The survey was conducted between March/April and September/October, 2015.

A grid based occupancy approach was used to estimate the presence/absence status of red pandas in the study zone. Presence/absence data were analyzed using presence/absence site occupancy models for occupancy estimation in PRESENCE Version 10.9_160420 [28]. The data were recorded into program PRESENCE as 1 and 0 representing detection and non-detection respectively within the grid. These data were used for estimating detection probability (p) and site occupancies (Ψ) as described by MacKenzie et al. [29]. Custom Model was then used for a single season analysis and two models were pre-defined to estimate the occupancy.

- 1 group, constant p: This model assumes that red pandas are detected with a single probability [p] in all the sampling grids.
- 1 group, survey-specific p: This model assumes that the detection probability of red pandas at all sites varies during different survey efforts.

PRESENCE ranks all models with the same data set according to the Akaike’s Information Criterion (AIC) [30]. AIC is a numerical ranking tool for model outputs and the lower its value the better the model–fits the data provided [31].

The Maximum Entropy Modeling–MaxEnt [32] was used to determine predictive spatial distribution of red pandas using MaxEnt version 3.3.3k. The red panda presence data and environmental parameters were used for this assessment. Geographic coordinates of red panda presence location (n = 125) collected during the field survey were used as presence data. Similarly, altogether 19 bioclimatic variables including 11 temperature and eight precipitation metrics along with altitude, slope, aspect, land cover and NDVI were used as environmental parameters for developing a distribution model. Multicollinearity analysis was also performed using MASS package and fmsb in R which revealed that all other factors except elevation, aspect, slope, bio3, bio4, bio8, bio16 and NDVI were collinear while taking Variance Inflation Factor (VIF) = 10 as a threshold. A set of 19 bioclimatic layers and altitude were obtained from WorldClim–Global Climate Data (Version 1.4), whereas the land cover data developed by ICIMOD with eight classes (e.g. forest, shrub, grass, bare area, agriculture, built up, water body and snow) were used. The NDVI was obtained from Land Sat 5, 2010.

All variables were converted into the format of ascii raster images with a cell size of 30 arc seconds (~1 km) clipped by the CHAL boundary. We set the program to run 5000 iterations with a convergence threshold of 0.00001, a regularization multiplier of 1, a maximum number of 100,000 background points, the output grid format as “logistic,” algorithm parameters set to auto features, and all other parameters at their default settings. Random test percentage was considered to be 25% of presence locations to test the performance of model. The model was run several times until the probability values of different variables were less than 1 which were excluded in successive efforts. Finally, we ran a predictive distribution model in the MaxEnt using the 8 variables that significantly contribute to red panda distribution. Model performance was assessed by the Area Under Curve (AUC) of the Receiver-Operating Characteristic (ROC) plot and the preparation and analysis of spatial data were performed using ArcGIS 10.3.

Red pandas are highly elusive animals, so it is hard to estimate their population size through direct sightings. Therefore, the estimation of encounter rate of their signs was used as a standard method to measure their relative abundance [25]. This was calculated by estimating the Encounter Rate (ER) of red panda signs per km within the survey grids.

### Habitat use

We established a concentric sampling plot of 10 m radius at each record site and collected data on elevation (m), substrate, vegetation types, average bamboo height, culm number, and distance to the nearest livestock shed, settlement, and water sources from each plot [27].

To determine vegetation types, we set up 968 concentric plots at every 500 m and 135 plots on record site [27]. The plots differed in radius: 10 m for trees (plot size 314.28 m^2^), and 3 m^2^ for bamboo (28.28 m^2^). The information on landscape level covariates including aspect, slope, NDVI (Normalized Differential Vegetation Index), canopy cover and distance to nearest village was based on GPS coordinates were retrieved by using ArcGIS 10.2 Version while the distance to the nearest livestock herding station was estimated based on visual estimation.

Likewise, we considered elevation, aspect, slope, proximity to water sources, and use of substrate for defecation for habitat use analysis. We also took into account vegetation analysis in terms of Importance Value Index (IVI) of trees and bamboo cover as well as height and density [31].

Statistical tests were performed to analyze the significant role of habitat variables. Nearly 37.5% (n = 411) of sampling plots (both sign and non-sign) were randomly chosen for performing non-parametric bootstrapped Welch t-tests (n = 99) to identify the distribution of t-statistics, associated p-value, and mean of variables across red panda presence plots. We performed X^2^-test and Fischers’-test to analyze the significance of aspect and bamboo cover in red panda distribution.

## Results

### Local names, socio-cultural and religious use

Based on the consultation with local people (n = 242), they were found to be using different names for the red panda in different district as follows:

Lamjung: Meta Sayal, Leta Sayal and Cherrah
Gorkha: Paamsyang
Manang: Wah and Lheete
Kaski: Nyakarau
Nuwakot: Machyang
Myagdi: Okra
Rasuwa: Hope
Dhading: Khop and Panichha

Members of the Chantyaal ethnic group of Gurjakhani Village Development Committee (VDC), Myagdi district consider red panda to be protective animals. Their shamans (traditional healers) use red panda hides as ritual dress while treating the sick (Second author, Pers comm. 2015). Similar beliefs were also reported to be common amongst the Magar ethnic group of Lamjung district. Pseudonyms of respondents were recorded on the datasheet to maintain their anonymity.

### Occupancy, distribution and abundance

Red pandas have been documented in five different districts: Rasuwa, Myagdi, Baglung, Nuwakot, and Dhading. A total of 132 indirect signs and 3 direct sightings were recorded in those five districts. Direct sightings were limited to the Ghyangphedi Buffer Zone of LNP in Nuwakot district. Occupancy of red panda was previously documented in the first four districts, while a reported presence in Dhading district was for the first time. However, the present study did not record any signs in the previously confirmed Manang and Gorkha districts.

An average encounter rate of 0.38 signs/km was recorded in CHAL with Nuwakot District having the highest rate (ER = 2 signs/km) followed by Myagdi (ER = 1.83 signs/km) and Rasuwa (ER = 0.36 signs/km) districts (Table 1).

**Table 1:** Relative abundance of red panda in CHAL.

Districts	No. of sign	Transect surveyed (no.)	Length of transect (km)	ER (signs/km)
Dhading	3	22	18.92	0.16
Baglung	1	71	49.68	0.02
Nuwakot	26	10	13.00	2.00
Rasuwa	14	46	39.10	0.36
Myagdi	81	59	44.25	1.83
	125	378	332.64	0.38

Red Panda’s naïve occupancy was estimated to be around 0.0667. Occupancy probability was modeled in two different models (Table 2), and the standard occupancy model with lower AIC showed an average occupancy probability for red panda to be around (0.06±0.01) with detection probability of 0.44±0.03. It indicated that nearly 7% of the total potential red panda habitat i.e. 218 km^2^ was occupied with red panda in CHAL. Similarly, an average detection probability of red panda in CHAL was estimated to be around 0.4482±0.0377.

**Table 2:** Model comparison for detection probability.

Model	AIC	ΔAIC	AIC weight	Model Likelihood	no. of parameter	Deviance
psi(.),p(.)	363.66	0.00	0.9985	1.0000	2	359.66
1 group, Survey-specific P	376.61	13.95	0.0015	0.0015	13	352.61

**Fig 3:**
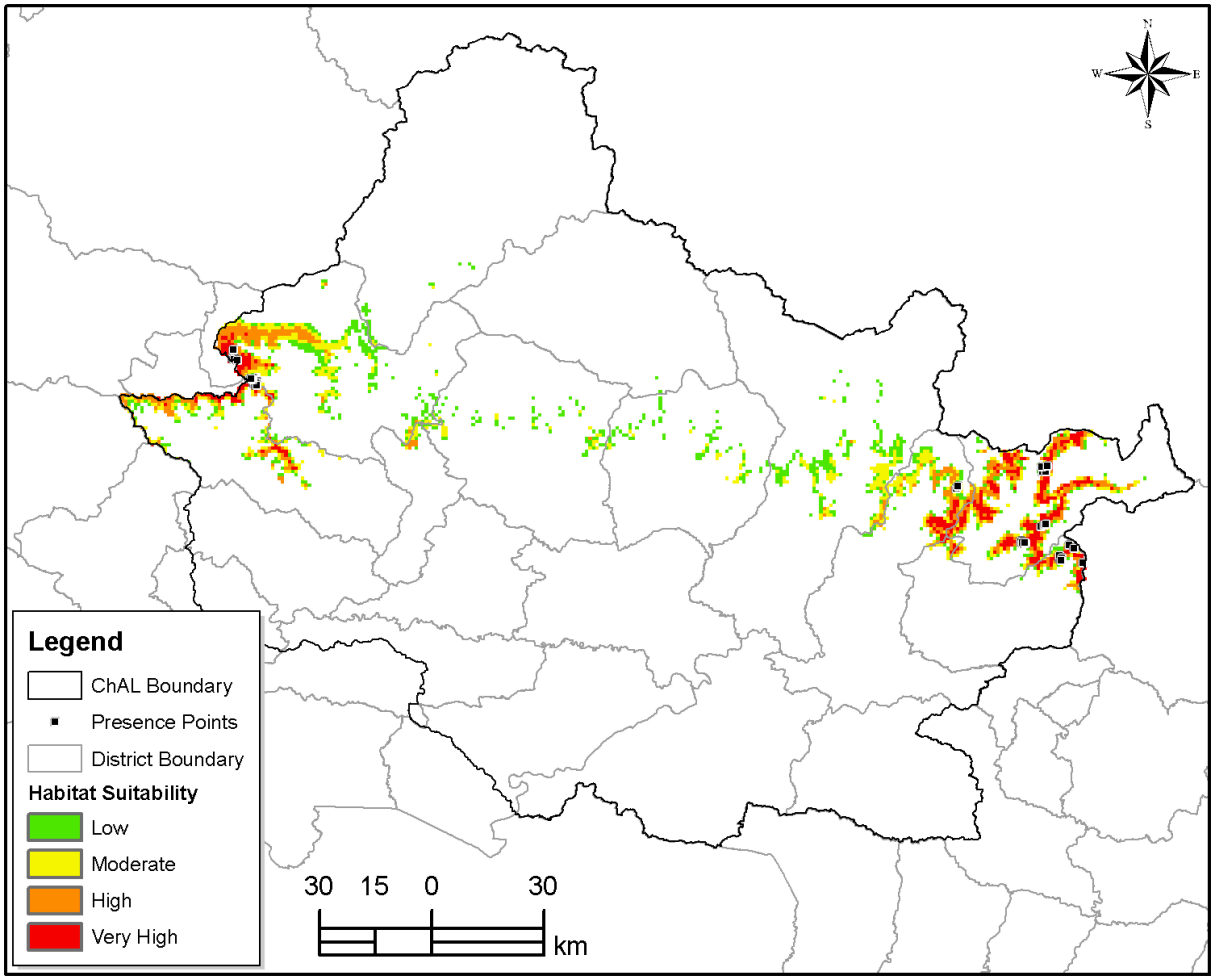
Potential red panda habitat with suitability status.

Predictive distribution model developed in MaxEnt with an average test AUC value of (0.994±0.002) at 0.5 thresholds indicated nearly 1,904.44 km^2^ of potential red panda habitat available in CHAL. The majority of the habitat falls in two extreme end of this landscape with a very small proportion in the central region (Fig 3). The district wise assessment of habitat availability indicated that Rasuwa had the largest potential habitat (26.06%) followed by Myagdi (23.91%) and Baglung (13.52%) respectively (Table 3). Similarly, the protected area covered nearly 41% of the total predicted habitat with the LNP having the highest contribution (69%) followed by ACA (26%), Manaslu Conservation Area (3%) and Dhorpatan Hunting Reserve (2%).

**Table 3:** Potential red panda habitat available in different districts.

Districts	Area (km^2^)	Percentage
Baglung	257.51	13.52
Dhading	210.06	11.03
Gorkha	209.28	10.99
Kaski	72.26	3.79
Lamjung	68.00	3.57
Manang	1.40	0.07
Mustang	18.45	0.97
Myagdi	455.40	23.91
Nuwakot	96.56	5.07
Parbat	19.21	1.01
Rasuwa	496.31	26.06
Total	1,904.44	100.00

The habitat suitability analysis was also performed by categorizing the potential habitat into four categories based on the probability of occurrence, which indicated that 16.58% (A = 315.81 km^2^) of the total potential habitat was highly suitable (probability value >0.5) and nearly one third of the total predicated habitat belonged to the low suitable category (probability value <0.1) category (Table 4).

**Table 4:** Red panda habitat suitability classes with available area in CHAL.

Suitability class	Probability value	Area (km^2^)	Percentage
Low	<0.10	578.86	30.40
Moderate	0.1–0.25	562.85	29.55
High	0.25–0.50	446.92	23.47
Very High	>0.50	315.81	16.58
Total		1,904.44	100.00

### Habitat use

Red Panda droppings (n = 179 piles) were observed on four different substrates: tree branches, fallen logs, ground, and rock. Trees were most common for defecation (62.21%) followed by the ground surface (29.96%) and fallen logs (8.70%). Rocks were the least preferred substrate.

Red Panda signs/sightings were recorded within a range of 190 m to 8000 m in distance from settlements with the majority (35.2%) recorded within a distance of 2000 m. Similarly, Red panda signs were observed between 35 m and 3000 m from herding stations though most of the signs (80.8%) were observed within 1000 m from livestock herding stations. The average distance to a herding station was 569.45±491.87 m.

**Fig 4:**
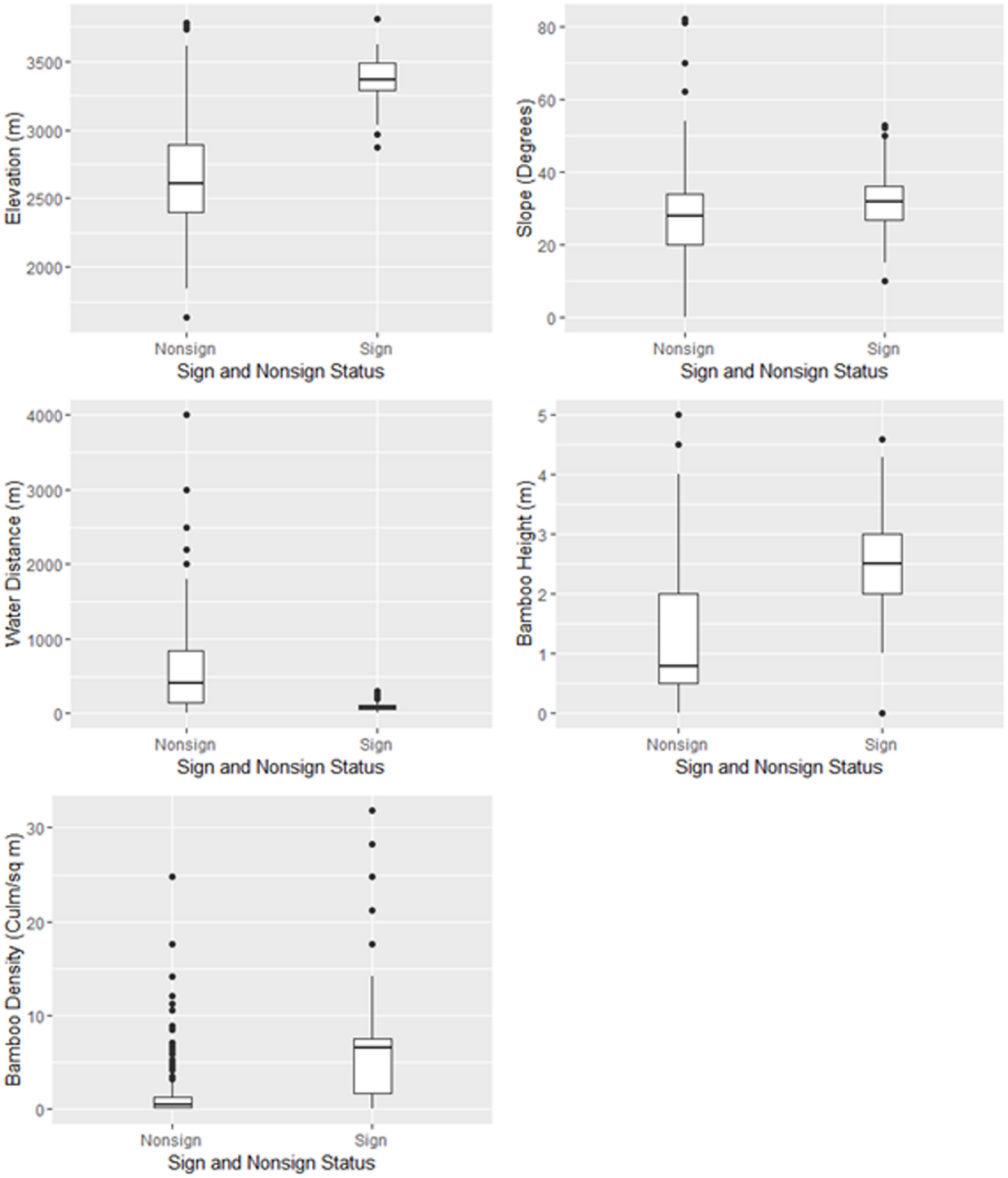
Box plot comparing the habitat variables in sign and non-sign plots.

Evidences of red pandas were observed between 2876 m to 3806 m elevation with an average elevation of 3380.38 m±138.49 m demonstrating its significant contribution to red panda distribution (non-parametric bootstrapped Welch t-test:-27.84±1.63, *p*~0.00). An elevation range of 3251 m to 3500 m was most preferred (61.6%), while a higher elevation (3501 m to 3750 m) was moderately preferred (22.4%). Red panda signs were found within a distance of 2 m to 300 m from water sources with an average distance of 90.66±58.82 m. The majority of signs (73.50%) were observed within 100m of water sources. None of the signs were observed beyond 300m from water sources, indicating the import of proximity to water sources for red panda distribution (non-parametric bootstrapped Welch t-test: -13.42±0.69, *p*~0.00),). Likewise, red pandas showed their preference towards slope 31.56°±8.47° with significant contribution in red panda distribution (non-parametric bootstrapped Welch t-test: -3.38±9.98, *p~0*.*00* as the majority of signs (50.4%) were recorded within the slope range of 31°-45° (Fig 4).

We also observed that red pandas showed a preference towards North-East aspect (32.8%) followed by Northern (23.2%) and Eastern (15.2%) aspects. Their least preference was observed in Southern and Western aspects indicating the significant role of aspect in red panda distribution (X^2^ = 6293, *df* = 7, *p* = 3.915e-1)

A total of 8598 trees representing 165 species were recorded in the survey area. Interestingly, only 25 species of tree were documented from red panda sign plots with the dominance of *Betula utilis* (*IVI* = 76.87) followed by *Rhododendron spp*. (*IVI* = 69.30) and *Abies spectabilis* (*IVI = 68*.*22*). Bamboo was found to be highly significant in red panda distribution as bamboo was recorded in nearly 89% of the sign plots, with density ranging from 0.21 to 31.81 bamboo culm/m^2^ and an average value of 6.01±5.59 culm/m^2^ (non-parametric bootstrapped Welch t-test: -7.80±0.96, *p*~0.00. Likewise, bamboo height ranged from 1 m to 4.6 m with an average value of 2.47±1.02 m indicating significant association in red panda distribution (non-parametric bootstrapped Welch t-test): -13.50±1.56, *p*~0.00. Similarly, the bamboo cover was also observed to be positively associated with one of the major factors influencing red panda distribution (Table 5).

**Table 5:** Variables influencing red panda distributions.

Variables	Test Types	Mean	SD	P
Elevation (m)	Welch t-test	3380.38	138.49	0.00
Slope (degree)	Welch t-test	31.56	8.47	0.00
Distance to water sources (m)	Welch t-test	90.66	58.82	0.00
dBH of the tree (cm)	Welch t-test	64.11	47.23	0.00
Tree height (m)	Welch t-test	13.22	6.36	0.00
Tree canopy cover (%)	Fischers’-test	48.87	19.53	0.00
Bamboo density (culm/m^2^)	Welch t-test	6.01	5.59	0.00
Bamboo height (m)	Welch t-test	2.47	1.02	0.00
Bamboo canopy cover (%)	Fischers’-test	41.15	21.79	0.00

## Discussion

### Current distribution in CHAL

This study has provided vivid information on the red panda status in the CHAL. Based on extensive ground truthing, red panda occupancy has been documented from only 7% (A = 218 km^2^) of the total potential habitat (A = 1904.44 km^2^) available in this landscape. Habitat loss and fragmentation have been observed to be as the prime challenge for red panda conservation. Besides, an indication of extirpation of red panda from some of the previously reported area indicates towards urgent need of conservation action to be taken to reverse the scenario.

The presence of red panda has been reported in Rasuwa, Nuwakot, Dhading, Myagdi, and Baglung districts. Jnawali et al. [17] also documented the presence of red pandas in these districts. Red pandas have previously been documented in Myagdi and Baglung districts [21,23] and Rasuwa district [5,7,17,20,33,34].

The present study failed to provide evidence that supports previously reported sightings in Dharapani and Thoche VDCs of Manang district [35,36] and Sirdibas VDC of Gorkha district [17]. Previously reported sites in Dharapani VDC were revisited and the habitat was observed to be entirely degraded because of a mass dyeing off bamboo following a forest fire. Heavy mortality of giant panda (n = 355) which also predominantly feeds on bamboo due to starvation because of natural collapse of bamboo population was also witnessed in China from 1971 to 1985 [37]. The previously reported site in Thoche VDC could not be visited during the survey. Likewise, consultations with local residents in Sirdibas VDC, Gorkha indicated that the red panda was present in their forest in the past. The last sighting in the areas was in 2014, when a red panda was caught in a trap targeted for pheasants. Evidence of red pandas also remained unreported from Mustang district with relatively good habitat in Lete VDC. Similarly, no evidence of red pandas was reported in Kaski district. Field surveys indicate that tourism fueling developmental activities have led to habitat loss and degradation, although very good quality habitat has been recorded in Ghandruk and Lwangghalel VDCs. Dorji et al. [38] have also indicated tourism as a cause of red panda habitat loss and degradation in Bhutan. Use of specific names in local dialects and ethno-zoological use of red panda hides indicate the presence of red pandas in the past in the entire CHAL. The fragmentation resulting in a number of tiny habitat patches might have not supported their presence in some of those areas as population falls below viable levels and extinction is ensued once the fragment gets smaller [39]. These observations stipulate the need of contiguous habitat to maintain the genetically viable population which could be done through the corridor development to link those isolated habitat patches. Besides, habitat management through the conservation of at least two different native bamboo species within red panda habitat is equally necessary so that one species compensates the loss of another species of bamboo during the mass flowering as length of flowering cycle varies with each species [40]. (Based on the occupancy modelling, the total habitat occupied by the red panda is estimated to be 218 km^2^. If we consider the ecological density of one red panda to be 2.9 km^2^ [40], then the population of red panda in the CHAL is 75 individuals. However, it may not be logical to generalize the ecological density established in 1991 with the current situation, though this population falls within the range estimated by Jnawali et al. [17] in 2012.

The relative abundance (*ER* = 0.38 signs/km) was reported to be lower than in the Sacred Himalayan Landscape of Nepal where an average *ER* was observed to be 0.87 signs/km. Record of presence from only few areas during the survey would have been resulted this low abundance as it has been generalized to the entire study area. Otherwise, the site specific relative abundance is more or less similar to previous studies from other areas in Nepal [24,41].

Probability of direct sighting of red panda during the survey was very low i.e. 2.4%. There were only 3 sightings while the indirect signs i.e. droppings were observed in 132 different locations. Williams [6] also reported similar experience in Ilam, Eastern Nepal where direct sightings were made in five different occasions with one additional case of dead body during 67 field days. Similarly, Bhatta et al. [42] also spotted red panda in only one occasions out of 28 indirect signs observed. Out of 179 dropping piles examined in the field, the population structure was found to be comprised of adult and cubs representing 81.82% and 18.18% respectively. This observation showed an evidence of reproductive population surviving in study area. Evidence of both adult and cubs were observed in Myagdi, Rasuwa and Dhading districts. Whereas, the sign of only cub was recorded in Baglung district though it was also an indication of the presence of mother. In contrast, no evidence of cubs were observed in Nuwakot district in spite of the record of a large number of dropping piles of adults (n = 25). The survey time might have influenced this observation as the survey in first 4 districts was carried out after the birth season (September/October) while the survey in Nuwakot district was conducted during re-birthing season (March/April) [9].

### Influence of macro-habitat use

Our study, based on MaxEnt modeling, indicated a very small proportion (16.58%) of available habitat occupied by the red panda in CHAL falls into the highly suitable category. The majority of the habitat is available in two extreme ends of this landscape with a very small percentage in the central region. The lower suitability in the central region is attributed to increasing anthropogenic influences resulting in habitat loss and degradation [6,20,38]. Less precipitation accounts for the lower quality habitat in Manang and Mustang districts as their location lies on the leeward direction with minimal annual precipitation. Out of the 8 variables considered for developing predictive distribution model, seasonal precipitation was observed to be one of the most significant variables affecting distribution. This observation supports the finding of Yonzon et al. [33]. Besides, loss of habitat due to a mass flowering of bamboo followed by a forest fire in previously occupied areas also has been a key factor [43]. Williams et al. [43] and Sharma et al. [44] had also attributed the vulnerability of red pandas to bamboo loss.

Trans-human practices are also detrimental for red panda conservation as livestock share red panda habitat during April to October every year. This creates competition for food resources as the livestock also prefer to feed on bamboo, the major diet of red pandas. In addition, demand for fodder, firewood, and timber to construct and maintain herder sheds contributes to deforestation and habitat degradation [43]. Observation of tree stumps (105.27/ha) and looped trees (135.16/ha) shows active deforestation and habitat degradation around these herder sheds. The dogs these herders use to guard their livestock sometimes kill red pandas and other associated wildlife [43,44,45]. The transfer of livestock diseases to red pandas is also a danger [46].

This study shows that a majority of suitable habitat (71%) falls outside current PAs. Conservation status outside the PA is relatively poor with respect to the scenario inside the PA. Forest biodiversity outside the PA is threatened mainly by deforestation and forest degradation through land-use conversion for agriculture, illegal settlements, infrastructure (including roads and electric transmission lines), and actions relating to the use of resources including overgrazing, unsustainable exploitation of forest products, habitat fragmentation and uncontrolled forest fires [27]. All those drivers of habitat loss and fragmentation observed in the CHAL have further deteriorated the habitat quality outside the PAs. Furthermore, the weak presence of government bodies outside the PAs aggravates threat level. Consequently, conservation efforts in CHAL will depend on effective management that balances conflicting needs of people and maintenance of biodiversity.

The red panda is an arboreal mammal which spends most of its time on trees, foraging and resting [5]. Observation of their dropping piles during this study also showed they spent most of their time on trees which might be because of their defense mechanism to avoid predators on the ground and to bask in the sun. But, they were also reported to be occasionally on ground which can be explained by their need for water and bamboo shoots especially during the monsoon season [5].

The presence of red pandas within an altitude range of 2876 m to 3806 m observed in our study is similar to the finding of Yonzon et al. [33]. In addition, this study supports previous studies [4,5,38] that have concluded that proximity to water sources is one of the important habitat requirements, based on observing more than 70% of red panda droppings within a range of 0 to 100 m from water sources. This is important to supplement the low water content associated with bamboo leaves [38,47]. Besides, this might be for avoiding predators and conserving vital energy while walking a long distance for water.

Contribution of slope and aspects were also found to be influential in red panda distribution. An average slope or 31.56°±8.47° was reported to be preferred in our study which is almost similar (34°±16°) to another study in Bhutan [38]. But this observation contradicts with the finding of a study in China which shows the preference more than 45° slope, and avoids areas with slopes of 15° to 30° [5]. Most of their evidences were observed in north-eastern, northern aspects, which is quite similar to other two studies [33]. Their preference towards these aspects may be because of availability of more food and water unlike in the southern and eastern aspects where the micro-habitat condition could not be supportive for abundant growth of bamboo; water availability and canopy cover [38].

Vegetation composition was also observed to have a pronounced effect in red panda distribution. Forests dominated by *Betula utilis*, *Rhododendron spp*. and *Abies spp*. with the bamboo in understory were observed to be prominently preferred by red panda. Preference towards these species was also reported in previous studies from central and western Nepal [21,38]. This is probably because of a number of other habitat variables contributing favorable conditions for red panda survival in these particular forests. However, this finding is dissimilar to findings in eastern Nepal and Singhalila in India [38,44] where observations show that red pandas prefer broad-leaf deciduous and sub-alpine forests.

A good quality canopy provides better shelter and safety from predators and easy movement from the branches of trees [4,47]. Likewise, preference towards the forest with higher dBH is probably because these large trunk trees provide the facilities for resting, nesting, and escaping from predators [6,20,28]. The presence of bamboo observed in 89% of sign plots demonstrates the importance of bamboo as one of the fundamental parameter affecting their distribution [6,20,26,38]. This is crucial, as bamboo leaves and shoots together constitute 83% of the red panda diet [20]. Moreover, the use of tree branches for foraging bamboo above the ground helps them avoid encounters with some predators [4,38,46].

## Conclusion

This is the only study conducted so far in Nepal that covers such a large area with extensive field work to collect empirical data and anecdotal information on red pandas. The study provides a vivid overview of the status of red pandas in the CHAL. While we have not been able to confirm the presence of red pandas previously documented in certain locations, we have noted a presence in new areas. The former might be a result of insufficient research effort in previously reported areas, especially Dharapani and Thoche VDCs in Manang district and Sirdibas VDC in Gorkha district. The absence of red pandas in previously reported areas indicates a need for immediate action to ensure the conservation of this species in CHAL. Besides, this work also provides further avenues to carry out an in-depth study on the impact of climatic and non-climatic environmental factors on red panda distribution and survival in CHAL which is crucial for devising an appropriate conservation long-term plan.
